# Supplementary material for: Cannabigerol Modulates Cannabinoid Receptor Type 2 Expression in the Spinal Dorsal Horn and Attenuates Neuropathic Pain Models
Source: Pharmaceuticals (Basel). 2025 Oct 8;18(10):1508. doi: 10.3390/ph18101508 (PMC12567403; doi:10.3390/ph18101508)
Supplement: Supplementary file 1 [file pharmaceuticals-18-01508-s001.zip › pharmaceuticals-3861955-supplementary.pdf]

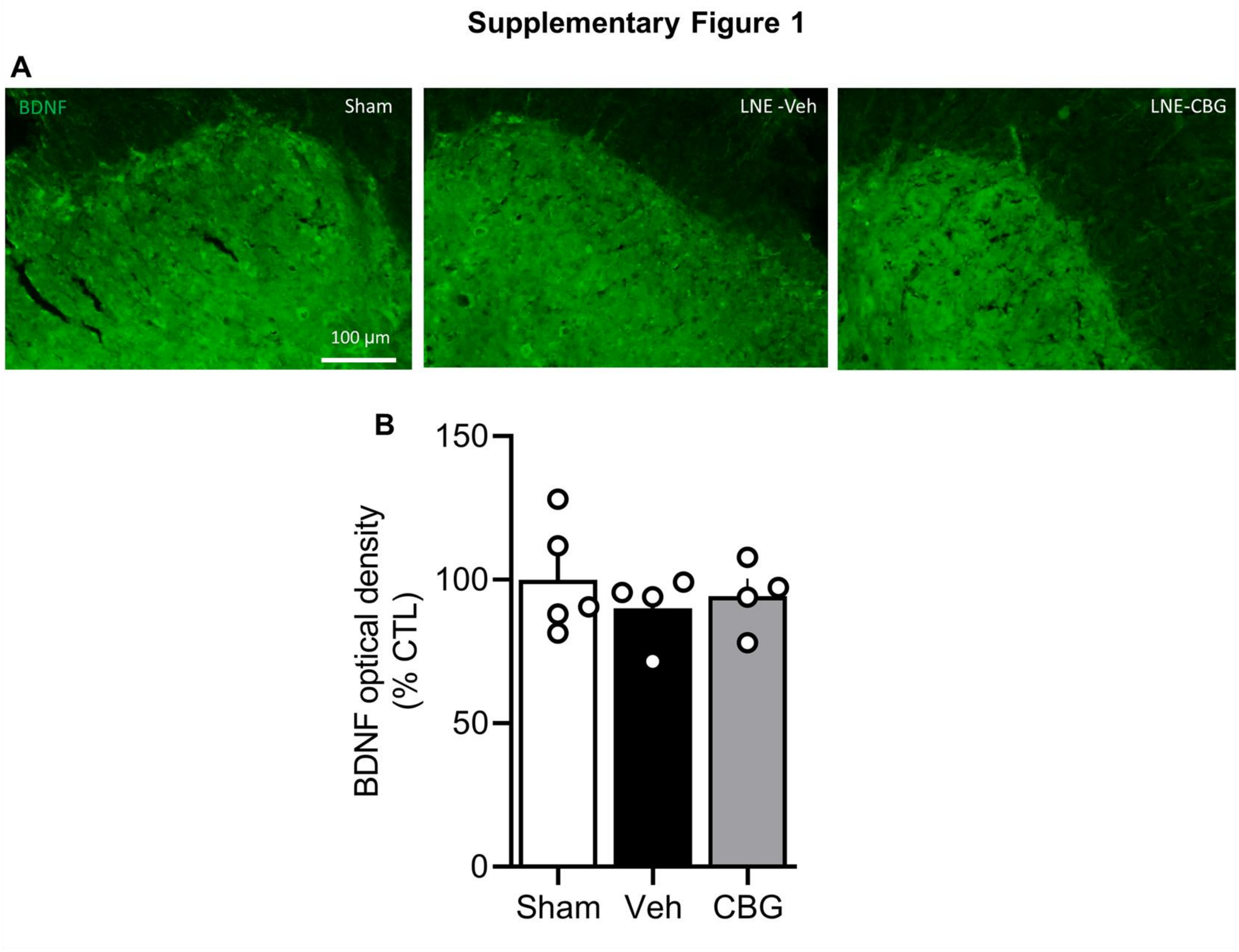

Supplement. Figure S1. Representative image of BDNF (A) in laminae I/II of the spinal cord dorsal horn. Neither SNL nor Cannabigerol had any effect on BDNF optical density (B; n=5,4 and 4, respectively). Graphs are expressed as mean ± SEM.
